# Is Drug Treatment for Dementia Followed Up in Primary Care? A Swedish Study of Dementia Clinics and Referring Primary Care Centres

**DOI:** 10.1371/journal.pone.0057161

**Published:** 2013-02-20

**Authors:** Lars Sonde, Kristina Johnell

**Affiliations:** 1 FOU nu - Research and Development Centre, Stockholm, Sweden; 2 Stockholm Gerontology Research Center, Stockholm, Sweden; 3 Aging Research Center, Karolinska Institutet and Stockholm University, Stockholm, Sweden; Oregon Health & Science University, United States of America

## Abstract

**Purpose:**

It is largely unknown how the medical treatment of patients diagnosed with dementia is followed up in primary care. Therefore, we studied patient medical records from two dementia clinics and from the referring primary care centres.

**Methods:**

A retrospective study of 241 patients was conducted from April to October 2011 in north west Stockholm, Sweden. Over half (51.5%) of the patients had Alzheimer’s disease (AD), the remainder had mixed AD/vascular dementia (VaD). Eighty-four medical reports from primary care (35% of the study group) were analysed at follow-up 18 months after diagnosis.

**Results:**

All four dementia drugs available on the Swedish market (three cholinesterase inhibitors [donepezil, rivastigmine and galantamine] and memantine) were prescribed at the two dementia clinics. The most commonly used dementia drug was galantamine. There were differences between the two dementia clinics in preference and combination of drugs and of treatment given to male and female patients. At follow-up, 84% were still on dementia medication. Drug use was followed up by the general practitioners (GPs) in two-thirds of the cases. Eighteen per cent of the GPs’ medical records made no reference to the patient’s dementia or treatment even though dementia drugs were included in the list of medications prescribed.

**Conclusions:**

The results indicate that the Swedish guidelines for treatment of cognitive symptoms in AD are being followed in primary care. However, documentation of follow-up of drug treatment was sometimes insufficient, which calls for development of guidelines for complete medical records and medication lists.

## Introduction

Dementia drugs provide symptomatic treatment and can affect cognition and global function in Alzheimer’s disease (AD). [1], [2] This effect is probably best achieved if the treatment is administered early, ideally immediately after cognitive examination and diagnosis. [3] Guidelines issued by the Swedish National Board of Health and Welfare for treatment of dementia state that patients with mild to moderate AD should be offered cholinesterase inhibitors for cognitive symptoms, while patients with moderate to severe AD should be prescribed memantine. The guidelines also declare that treatment must be followed up for dose adjustment and regularly thereafter at least once a year. [4]

Within the Stockholm health authority area, cognitive examinations are conducted by general practitioners (GPs) and at dementia clinics. Often, GPs refer patients to dementia clinics for specialist evaluation and then resume responsibility for patient treatment once the clinical examination has been completed.

There are currently four dementia drugs available on the Swedish market; three cholinesterase inhibitors (donepezil, rivastigmine, galantamine) and the NMDA receptor antagonist memantine. [5] The three cholinesterase inhibitors differ in terms of acetylcholinesterase and butyrylcholinesterase inhibition, pharmacokinetics, interactions and adverse reactions. [6], [7] Approximately two out of three patients who are treated with a cholinesterase inhibitor demonstrate a positive response to the treatment. [8] These drugs are usually well-tolerated and the main adverse drug reactions are gastrointestinal disorders. [9], [6] Memantine is a non-competitive NMDA receptor antagonist that can help to mitigate the cognitive symptoms and maintain global function. Again, the effect varies between patients and the drug dose should be adjusted in patient with impaired kidney function. [10]

It is largely unknown how the medical treatment of patients diagnosed with dementia is followed up in primary care. Therefore, the aim of this study was to investigate the medical treatment of patients who was examined and diagnosed in two dementia clinics and then referred back to their GPs.

## Methods

### Participants

A retrospective study was conducted from April to October 2011. All patients (n = 616) who had been referred to two dementia clinics for a cognitive examination in 2008 were included. Both clinics have their catchment area in north west Stockholm, Sweden. We then selected the patients who were diagnosed with AD or mixed AD/vascular dementia (VaD) and who were prescribed dementia drugs (cholinesterase inhibitors and/or memantine) (n = 331; 54%).

**Figure 1:**
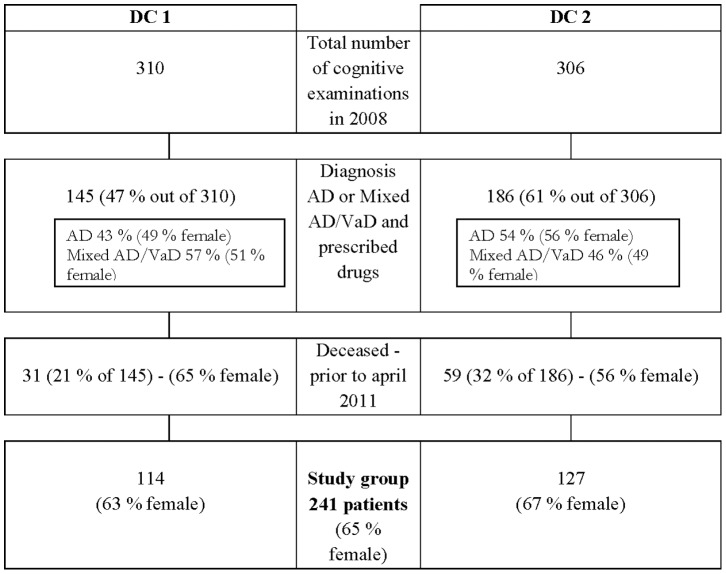
Flow chart describing the patients included in the study.

By April 2011, 90 (27%) of the patients registered in 2008 had died. The remaining 241 patients (the study group) received a letter from their dementia clinic informing them of the study and asking their permission to acquire their medical records from their GP. The letter also contained a consent certificate to be signed by the patient or a member of his/her family granting permission to access the patient’s medical record and returned to the clinic. Figure 1 shows the process of participant selection.

Table 1 shows the flow from study group to follow-up group. A total of 140 consent certificates were returned, after which the GPs responsible for 127 of the patients were contacted (13 patients were not registered with a GP). We then requested access to the patients’ medical records from the time of diagnosis to 24 months later. A total of 84 medical records were received from the GPs (35% of the study group).

**Table 1:** Patients from study group included in follow-up group and response rates.

	Number of patients	Number of patients	Total	%
	DC 1	DC 2		
**Study group**	114	127	241	
Consent forms	80	60	140	58
Letters to responsible GP	77	50	127	53
Patients not registered with a GP	3	10	13	
Patients not registered at listed GP	14	11	25	
No answer from GP	12	6	18	
**Follow-up group**	51	33	84	35

### Data collection

The following variables were registered from the medical records obtained from the dementia clinics and GPs: age, sex, diagnosis, type of dementia drug (at start and at follow-up), residential status (regular accommodation or nursing home). For the follow-up group, it was also noted whether the drug was registered on the prescribed medication list and whether the GP or dementia clinic followed up the treatment. All information relevant to the drug treatment was also recorded.

### Analysis

The results are presented by group (study group and follow-up group) and, where necessary, by clinic (dementia clinic 1 (DC1) and dementia clinic 2 (DC2)). Comparisons between clinics were made using Student’s t-test and Fisher’s exact test.

### Ethical concerns

This study was approved by the regional ethical review board in Stockholm (dnr 2011/71-31/1).

## Results

The average age among the 241 dementia patients was 81 years and 65% were women (Table 2). There was no difference in average patient age or gender distribution between the two dementia clinics. The distribution of AD and mixed AD/VaD varied, with a higher proportion of the latter at DC1 and of the former at DC2. These differences were not statistically significant. There were, however, significantly fewer men (p = 0.03) who had received an AD diagnosis at DC1 compared with DC2 (33.3% and 59.5%).

**Table 2:** Baseline data for patients from dementia clinics and prescribed dementia drugs.

	Total
**Number of patients**	241
**Mean age**	81.1 (±6.0)
**Range**	65–94
**Male/female (%)**	84/157(35/65)
**AD / Mixed AD/VaD %**	51.5/48.5
**AD / Mixed AD/VaD % Male**	46.4/53.6
**AD / AD+VaD % Female**	54.1/45.9
**Prescribed dementia drugs:**	
**Galantamine (%)**	109 (45.2)
**Rivastigmine (%)**	41 (17.0)
**Memantine (%)**	28 (11.6)
**Donepezil (%)**	25 (10.4)
**Galantamine and Memantine (%)**	32 (13.4)
**Memantine and Rivastigmine (%)**	4 (1.6)
**Donepezil and Memantine (%)**	2 (0.8)

### Dementia drugs

All four approved dementia drugs were prescribed at the two dementia clinics (Table 2). Galantamine was the most common drug. The proportion of prescriptions of memantine, particularly in combination with galantamine, varied between the two dementia clinics. DC1 prescribed this drug in 47.4% of the patients, while DC2 prescribed it only sporadically (7.8%). On the other hand, donepezil was prescribed to a fifth of the patients at DC2 (20.5%) and only to a small minority of patients at DC1 (0.9%). Prescriptions of rivastigmine also differed between the two clinics (DC1 7.9%, DC2 27.6%).

Galantamine was used for AD and mixed AD/VaD, and was prescribed for almost half of the patients in the study group (Table 3). At DC1, the combination of galantamine and memantine was the preferred prescription for men with AD, while at DC2 it was either galantamine or rivastigmine. Men with mixed AD/VaD tended to be given galantamine at both clinics. At DC1, galantamine was combined with memantine as an alternative, while DC2 had rivastigmine as second choice (Table 3).

**Table 3:** Prescription of dementia drugs from dementia clinics to patients according to specific diagnosis and gender.

DC 1 (n = 114)				DC 2 (n = 127)		
Prescribed drugs				Prescribed drugs		
AD	AD+VaD				AD	AD+VaD
Male/Female	Male/Female	%		%	Male/Female	Male/Female
29% / 46%	54% / 39%	**44**	**Galantamine**	**46**	40% / 49%	41% / 51%
21% / 13%	11% / 27%	**17**	**Memantine**	**7**	4% / 4%	6% / 10%
50% / 31%	25% / 15%	**28**	**Galantamine and Memantine**	**1**	0% / 0%	0% / 3%
0% / 5%	4% / 18%	**8**	**Rivastigmine**	**25**	40% / 22%	35% / 15%
0% / 2.5%	7% / 0%	**3**	**Memantine and Rivastigmine**	**1**	0% / 0%	18% / 3%
0% / 2.5%	0% / 0%	**1**	**Donepezil**	**19**	16% / 22%	0% / 18%
-	-	-	**Donepezil and Memantine**	**1**	0% / 2%	
100/100	101/99	101	**Total**	100	100/99	100/100

Women with AD were prescribed galantamine as first choice. At DC1, memantine and rivastigmine were used as second choice, while DC2 preferred donepezil. A similar pattern was seen for women with mixed AD/VaD, although memantine and rivastigmine were the second choice drugs at DC1 (Table 3).

### Follow-up

A total of 84 medical records were received from the GPs. The average age of the follow-up group was 84 years. Six out of ten were women and nine out of ten lived at home. Forty-three patients (51%) had received an AD diagnosis (Table 4).

**Table 4:** Follow-up group: Demographic data and follow-up responsibility.

	Total
Number of patients	84
Mean age Range	(80.4±6.4) 65–94
Male/Female(%)	35/49 (42/58)
AD diagnosis (%)	43 (51)
Regular accommodation (%)	76 (91)
Nursing home	8
Patients still on dementia drugs (%)	71 (84)
Drugs specified in medication list (%)	60 (84)
Followed up by GP (%)	47 (66)
Followed-up by dementia clinic (%)	22 (31)

Regarding the medical treatment, 71 patients (84%) were still on dementia medication at follow-up. Dementia drug treatment had been stopped in seven patients because of lack of effect or adverse reactions. For six of the patients, details about their medication were missing.

Fifteen of the medical records (18%) lacked any reference to the patient’s dementia or dementia treatment. In 16% of the medical records, the drug was not included on the accompanying medication list, but its use could be verified from the case notes.

The 71 patients who were still on medication were followed up by their GP in 66% of the cases. The dementia clinics regularly followed up drug treatment according to 31 per cent of the case sheets. In two cases, the patients were followed up by both their GP and the clinic. Conversely, in two other cases, there was no information about whether the dementia drug treatment was prescribed by the GP or at the dementia clinic (Table 4).

## Discussion

The results of this study show that a large proportion of primary care patients are still on the dementia medication initiated by the dementia clinic, indicating that the Swedish guidelines are being followed by GPs. [4], [11] However, many of the patients’ primary care medical records were incomplete regarding notes on follow-up of medical treatment and/or regarding the prescribed medication list. [12] These results indicate that healthcare authorities should provide clear guidelines on how to document follow-up of treatment.

There were differences in treatment between the dementia clinics in terms of drugs prescribed and of the patients’ sex. Previous studies have also found considerable differences between practices [13] and settings. [5], [14] Whether this reflects differences between the composition of dementia groups or therapy traditions should be investigated in larger studies with higher statistical power or with a qualitative approach.

The Swedish National Board of Health and Welfare recommends that cholinesterase inhibitors should be offered to people with mild or moderate AD and that memantine to people with moderate to severe AD. [4] A considerable difference was observed in the prescribing of memantine between the dementia clinics. The main difference between male and female patients was when galantamine and memantine was prescribed concomitantly.

Although several studies have demonstrated effects of both cholinesterase inhibitors [15], [16] and memantine [10] in AD, some argue that there is a big difference between the results from randomized controlled trials compared to what practitioners observe in the clinical setting. One Italian study examined almost one thousand people who were on one of the three available cholinesterase inhibitors and concluded that the patients deteriorated over time (36 months) and that there were no significant differences in efficacy between the cholinesterase inhibitors in terms of a set of functional and cognitive parameters. [17]

Adverse reactions are another possible reason why alternative drugs or combinations of drugs are tried. Galantamine and memantine, alone or in combination, were the most commonly used drugs at follow-up. Sixteen per cent of the patients who could be followed up had stopped taking their dementia drugs, owing, according to the case sheets, to a lack of effect and/or the magnitude of the adverse reactions. A Cochrane study from 2006 (based on 13 studies) established that cholinesterase inhibitors are efficacious for people with mild or moderate AD, but that no differences could be ascertained between the different cholinesterase inhibitors. Fewer adverse reactions were observed for donepezil than for rivastigmine. [6] However, these cholinesterase inhibitors were not frequently used in this study.

The low response rate at follow-up is a limitation of our study and affects the generalizability of the results. A possible reason for the low number of retrieved medical records is that it is difficult to obtain consent from dementia patients due to communication problems. [18] Another reason could be the different stages of the recruitment process. Initially, all patients were contacted by mail in order to provide information about the study and to obtain their consent to access their medical records from their GPs. This required a consent certificate to be signed and returned by the patients or a member of their families. At DC1 (where the response rate was higher), there was both time and opportunity to call the patients and remind them or their family member to complete the request. This was not possible at DC2.

It is also possible that the non-responders more often lived in nursing homes, [19] which means that responsibility for their medication might have been transferred from their GPs to the nursing home’s own doctor. Future studies should focus on this frail group, as considerable uncertainty exists regarding their dementia treatment and how it is followed up.

## Conclusion

Four out of five studied patients were still on their dementia drugs that had been initiated three years earlier by their dementia clinic. Our results indicate that the Swedish National Board of Health and Welfare’s recommendation to offer treatment for cognitive symptoms to people with AD is largely followed by primary care. However, documentation of follow-up of drug treatment was sometimes insufficient, which calls for development of guidelines for complete medical records and medication lists.

We also discovered that there was a wide variation between the dementia clinics as regards choice of drug and that there was a difference in drug treatment between male and female patients. These findings may indicate a need for better monitoring of drug treatment and implementation of evidence-based programs for prescribing of dementia drugs. In addition, this might reveal that there are gender differences in response and tolerance to dementia drugs among AD patients in clinical practice.
